# Stem/progenitor cells in closed suction drainage fluid after hip arthroplasty

**DOI:** 10.3109/17453674.2011.566153

**Published:** 2011-04-05

**Authors:** Satoshi Sera, Masakazu Ishikawa, Nobuo Adachi, Yuji Yasunaga, Mitsuo Ochi

**Affiliations:** ^1^Department of Orthopaedic Surgery; ^2^Department of Artificial Joints and Biomaterials, Graduate School of Biomedical Sciences, Hiroshima University, Hiroshima, Japan

## Abstract

**Background and purpose:**

Drainage after surgery is commonly used, and the contents are generally discarded as clinical waste. We analyzed closed suction drainage fluid from hip arthroplasty patients to determine whether any multipotent stem cells were present that could be used as a source of cells for tissue regeneration.

**Methods:**

Drainage fluid was obtained from 14 patients after hip arthroplasty on the day of surgery, the next day, and 2 days after surgery. Peripheral blood and bone marrow from the iliac crest were also obtained from the same patients during surgery. These samples were examined using regular flow cytometric profiling, and we performed quantitative immunoassays of stromal-derived factor-1 (SDF1) levels in the plasma. Mononuclear cells (MNCs) from these samples were also isolated and cultured. Fibroblastic adherent cells from MNC fractions were cultured in an osteogenic and a chondrogenic differentiation medium and were then evaluated for multipotentency.

**Results and interpretation:**

Fibroblastic adherent cells were isolated from the mononuclear cell fraction of bone marrow and drainage fluid on the day of surgery, but they were not present in either the mononuclear cell fraction of the peripheral blood or the drainage fluid on the next day and 2 days after surgery. The cells from the drainage fluid on the day of surgery could differentiate in vitro into osteogenic and chondrogenic cells. SDF1 was elevated on the day of surgery, while CXCR4 was elevated on that day and the next day. This suggests that locally-induced SDF1 contributes to the mobilization of circulating CXCR4-positive cells. These results show that the drainage fluid collected on the day of surgery contains stem/progenitor cells that could be used for autologous cell-based therapy.

Stem cells are vitally involved in tissue regeneration and homeostasis. Mesenchymal stem cells (MSCs) have the potential to differentiate into lineages of mesenchymal tissues, including cartilage, bone, fat, and muscle. Isolation and characterization of MSCs from bone marrow, muscle, adipose tissue, or synovial tissue have been reported (Pittenger et al. 1999, Lee et al. 2000, De Bari et al. 2001, Jones et al. 2002, Gimble et al. 2007).

The clinical use of MSCs is increasing, with profound implications for regenerative medicine. Several animal studies have demonstrated the efficacy of using stem cells in the treatment of bone defects (Ohgushi et al. 1989, Bruder et al. 1994, Ranieri et al. 2007, Veronique et al. 2007). Local injection of ex vivo expanded autologous bone marrow stem cells has been used successfully for the treatment of large bone defects in 3 patients (Quarto et al. 2001). Furthermore, with tissue engineering, a titanium-mesh scaffold filled with bone mineral blocks infiltrated with BMP7 and a bone marrow mixture were found to produce a large amount of bone tissue needed for reconstructing a mandibular defect in a patient (Warnke et al. 2004).

Drainage fluid connected to a cell-collecting device has been used for salvage of red blood cells to re-infuse after arthroplasty (Semkiw et al. 1989). White blood cells, including the MSC fraction, were usually discarded as clinical waste.

Stromal-derived factor-1 (SDF1) controls many aspects of stem cell function (Aiuti et al. 1997, Peled et al. 1999). The SDF1/CXCR4 axis plays an essential role in the mobilization and homing of stem/progenitor cells (Ceradini et al. 2004, Kucia et al. 2005). Furthermore, the expression of SDF1 is upregulated following irradiation and hypoxia, and SDF1 can induce the recruitment of endothelial progenitor cells in a regeneration model for myocardial infarction (Ceradini et al. 2004).Thus, it is of interest to investigate the mechanism underlying stem cell recruitment to injured tissues by evaluating the level of SDF1 or the frequency of CXCR4-positive cells in various samples.

We investigated whether the mononuclear cells (MNCs) in closed suction drainage fluid after hip arthroplasty could produce MSCs, and determined the level of SDF1 and frequency of CXCR4-positive cells in the drainage fluid to investigate the mechanism underlying stem cell recruitment to the injured tissue.

## Materials and methods

Informed consent was obtained from all patients. The research methods were reviewed and approved by the committee on human ethics of Hiroshima University.

After hip arthroplasty, the closed suction drainage fluid from 14 consecutive patients with a mean age of 66 (51–82) years was collected on the day of surgery, the next day, and 2 days after surgery. Peripheral blood and bone marrow from the iliac crest were also obtained during the surgery from the same patients using EDTA as anticoagulant.

### Immunophenotyping of cells

Peripheral blood, bone marrow, and closed suction drainage fluid were lysed using an ammonium chloride solution (STEMCELL Technologies Inc., Vancouver, Canada) for 10 min to remove red blood cells from the samples. After lysis of the red blood cells, the samples were centrifuged and washed twice with phosphate-buffered saline (PBS). The final samples were resuspended in 100 μL Hanks' Balanced Salt Solution (HBSS) (GIBCO Laboratories, Grand Island, NY) and stained with a combination of CD45-fluorescein isothiocyanate (FITC), CD34-phycoerythrin (PE), and CXCR4-allophycocyanin (APC) monoclonal antibodies (BD Biosciences Pharmigen, San Diego, CA) for 30 min at 4°C in the dark. Thereafter, the samples were washed twice with HBSS and resuspended in 500 μL HBSS for flow cytometric profiling with a FACSCalibur flow cytometer and the CellQuest software program (BD Immunocytometry Systems, Mountain View, CA). CXCR4+ cells were identified, and the percentage of CXCR4+ cells in MNCs was compared for each sample.

### Enzyme-linked immunosorbent assay for SDF1

The concentration of SDF1 in the samples from the last 5 patients was measured with an enzyme-linked immunosorbent assay using a kit (R&D Systems, Minneapolis, MN) according to the instructions of the manufacturer.

### Isolation and culture of cells

Closed suction drainage fluid was poured into a Leucosep 227290 (Greiner Bio-One Co. Ltd., Japan) with 15 mL separation medium Histopaque 1077 (Sigma-Aldrich, Oakville, Canada) and centrifuged at 1,000 *g* for 10 min. MNCs present in the enriched cell fraction were collected and washed twice with PBS, and the cell pellet was resuspended in Dulbecco's modified Eagle's medium (DMEM) (GIBCO Laboratories) containing 10% heat-inactivated fetal bovine serum (FBS) (Sigma-Aldrich) and antibiotics (penicillin 100 units/mL and streptomycin 100 μg/mL). 106 cells were seeded in a 100-mm diameter plastic dish and cultured at 37°C in a humidified atmosphere containing 5% CO2; the medium was changed on a weekly basis. Cell samples were cultured for approximately 3 weeks after the first seeding. Adherent cells were used in the following differentiation assays.

*Colony-forming-unit fibroblast assay.* The frequency of colony-forming-unit fibroblasts (CFU-Fs) was counted after culturing the MNCs for approximately 3 weeks in 60-mm diameter plastic dishes. The culture dishes were stained with Giemsa solution and cell clusters containing > 50 cells were scored as CFU-F colonies.

*Differentiation of adherent cells.* After culturing the MNCs for approximately 3 weeks, adherent cells were detached using trypsin solution (TrypLE Express, GIBCO) and 104 cells were seeded and cultured in a 60-mm diameter plastic dish for another 3 weeks for osteogenic differentiation using osteogenic differentiation medium (Poietics PT-3924; Lonza, Walkersville, MD). They were stained with 1% alizarin red S to visualize calcium deposition. The calcium content was measured using a Stanbio Total Calcium Liquicolor Kit (Stanbio Laboratory, Boern, TX). As a control group, additional cells were cultured with the standard medium throughout the experiment.

To assess chondrogenic differentiation, 2.5 × 10^5^ cells were placed in a 15-mL polypropylene tube and pellet culture was performed (three-dimensional culture) for 3 weeks using chondrogenic differentiation medium (TMDFC-001; TOYOBO, Japan). The differentiation was evaluated by histochemical staining with safranin O. The expression of 2 chondrocyte-specific genes, those encoding type-II collagen and aggrecan, was assessed by RT-PCR. From the pellet, total RNA was isolated using the RNAqueous Micro kit (Ambion, Austin, TX). First-strand cDNA was synthesized using Superscript III reverse transcriptase (Invitrogen, Carlsbad, CA) and amplified with Taq DNA polymerase (Amplitaq Gold; Applied Biosystems, NJ), according to the manufacturer's instructions. PCR products were analyzed by 1.5% agarose gel electrophoresis. The primers used were: human-specific GAPDH (596 bp): 5’-CTGATGCCCCCATCTTCGTC-3’ (forward) and 5’-CACCCTGTTGCTGTAGCCAAATTCG-3’ (reverse), human type II collagen (414 bp): 5’-CTGGCTCCCAACACTGCCAACGTC-3’ (forward) and 5’-TCCTTTGGGTTTGCAACGGATTGT-3’ (reverse), and human aggrecan (357 bp): 5’-CTACGACGCCATCTGCTACA-3’ (forward) and 5’-ACGAGGTCCTCACTGGTGAA-3’ (reverse). As a control group, a pellet was cultured with the standard medium throughout the experiment.

### Statistics

Data are presented as mean (SD). The paired t-test was used to assess calcium deposition and the Tukey-Kramer method for post-hoc testing was used to evaluate the differences in SDF1 plasma levels and the frequency of CXCR4 positive cells. Any p-value of < 0.05 was considered to be statistically significant.

## Results

### Immunophenotyping of cells by flow cytometry

**Figure 1.**
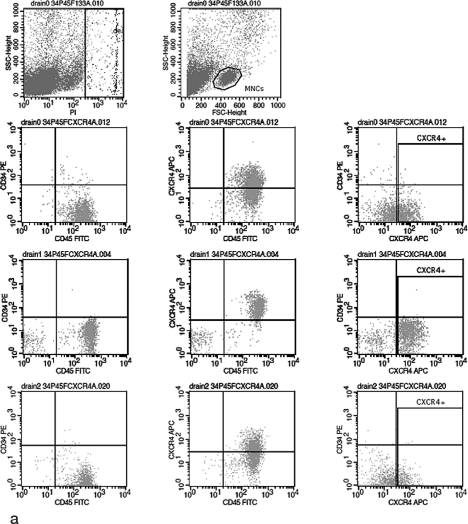

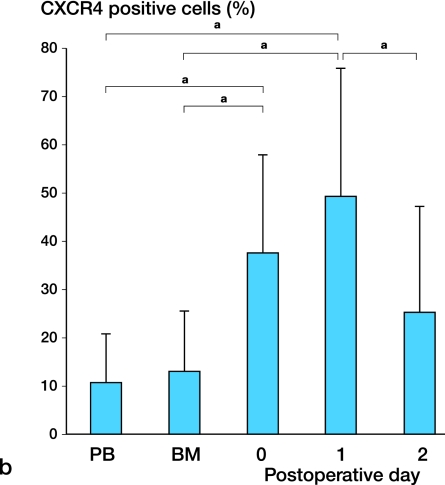
(a) Immunophenotyping results from a representative donor. Immunophenotyping of cells was confined to gated MNCs. Samples were stained with a combination of CD45-FITC, CD34-PE, and CXCR4-APC monoclonal antibodies. (Upper: the day of surgery; middle: the day after surgery: lower: 2 days after surgery). (b) The frequency of CXCR4-positive cells in MNCs was 11% (10) in peripheral blood; 13% (13) in bone marrow; and 38% (20), 50% (26), and 25% (22) in drainage fluid on the day of surgery (p.o.0 day), the next day (p.o.1 day), and 2 days after surgery (p.o.2 day).

Figure 1 shows the cell-surface antigen profiles of drainage fluid. The frequency of CXCR4-positive cells in the MNC fraction (on the day of surgery and the day after surgery in the drainage fluid) was statistically significantly increased in comparison to that in the peripheral blood and bone marrow. The frequency of CXCR4-positive cells was increased most on the day after surgery, and it was significantly increased in comparison to that in peripheral blood and bone marrow, and to that in the drainage fluid on the second day after surgery.

### Enzyme-linked immunosorbent assay for SDF1

**Figure 2.**
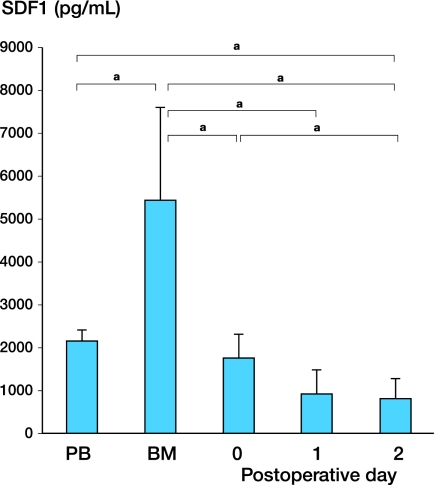
SDF1 plasma levels in the closed suction drainage fluid were 1,766 pg/mL (554), 942 pg/mL (574), and 828 pg/mL (460) on the day of surgery (p.o.0 day), the next day (p.o.1 day), and on day 2 after surgery (p.o.2 day). In peripheral blood and bone marrow, the SDF1 levels were 2,170 pg/mL (264) and 5,464 pg/mL (554), respectively.

The levels of SDF1 in the closed suction drainage fluid statistically were significantly elevated on the day of surgery in comparison to 2 days after surgery, and they were increased the most in the bone marrow (Figure 2).

### CFU-F assay

**Figure 3.**
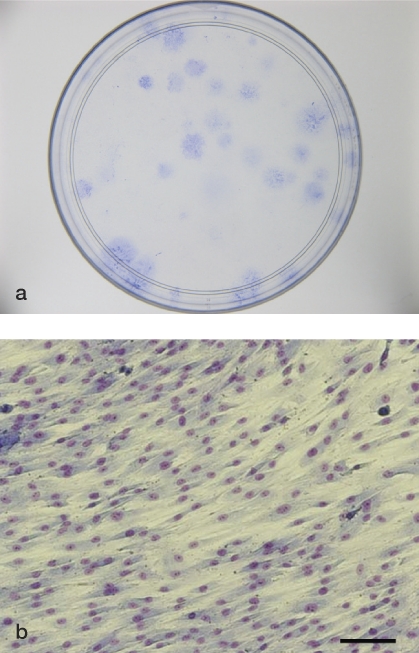
After culture, the MNCs from the day of surgery gave rise to colonies containing cells with a fibroblast-like spindle shape and these colonies resembled CFU-Fs. (a) 1×. (b) 20×. Bar represents 200 μm.

Table 1 shows the sample volume and the concentration and number of MSCs in the closed suction drainage fluid on the day of surgery, the next day, and 2 days after surgery. The MNCs obtained on the day of surgery gave rise to colonies showing a fibroblast-like spindle shape and these colonies resembled CFU-Fs (Castro-Malaspina et al. 1980). Culture of the MNCs for 3 weeks yielded 16 (5.2) CFU-Fs in the culture of MNCs from the day of surgery. In contrast, no adherent fibroblast-like cells or colonies could be detected in the cultures of the cells isolated from peripheral blood, or from the fluid collected on the next day or 2 days after surgery (Table 2, Figure 3a and b).

**Table 1.** MNCs in closed suction drainage fluid (n = 5). Mean (SD)

Days after surgery	Sample volume (mL)	MNC concentration (× 10^4^/mL)	Number of MNCs (× 10^6^)
0	300 (85)	11 (13)	30 (33)
1	150 (72)	4.9 (4.0)	7.3 (7.6)
2	51 (37)	6.8 (7.2)	2.4 (2.1)

**Table 2.** CFU-F numbers for each sample, after approximately 3 weeks of culture (n = 10)

Sample no.	From day 0	From day 1	From day 2	From PB	From BM
1	9	0	0	0	18
2	17	0	0	0	19
3	14	0	0	0	20
4	20	0	0	0	35
5	11	0	0	0	25
6	27	0	0	0	28
7	12	0	0		
8	17	0	0		
9	13	0	0		
10	21	0	0		

PB: peripheral blood; BM: bone marrow.

### Differentiation

**Figure 4.**
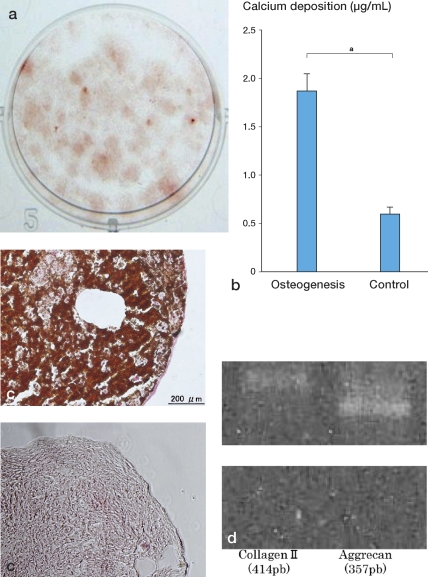
(a) Alizarin red S staining after 21 days of incubation in osteogenic medium (n = 4). (b) The calcium deposition assay showed the level to be 1.9 μg/mL (0.2) in cells cultured under osteogenic conditions and 0.13 μg/mL (0.04) in cells cultured in standard medium. (c) Safranin O staining (upper: chondrogenic medium; lower: standard medium) (n = 2). (d) RT-PCR analysis of RNA expression of the chondrocyte-specific genes encoding type-II collagen and aggrecan after 21 days of incubation (upper panel: chondrogenic medium; lower panel: standard medium) (n = 2).

Adherent MNCs were cultured under conditions favorable for osteogenic and chondrogenic differentiation. After 3 weeks of incubation under osteogenic conditions, the colonies were stained with alizarin red S to examine calcification (Figure 4a). Calcium deposition under osteogenic conditions was statistically significantly increased in comparison to that in cells cultured in standard medium (Figure 4b).

After 3 weeks of incubation under chondrogenic conditions, the cultured pellet stained positively with safranin O, but negatively after culture in the standard medium (Figure 4c). Expression of type-II collagen and aggrecan RNA was also detected under chondrogenic conditions, but it was not detected in the standard medium by RT-PCR (Figure 4d).

## Discussion

The identification of a feasible source of cells remains a challenge for tissue-engineering strategies (Pittenger et al. 1999, Lee et al. 2000, De Bari et al. 2001, Jones et al. 2002, Gimble et al. 2007). We found that osteogenic and chondrogenic progenitor cells could be isolated from the closed suction drainage fluid after THA. In the presence of lineage-specific induction factors, the adherent cells could differentiate in vitro into osteogenic and chondrogenic cells. However, the adherent cells were obtained only during the early phase after surgery. These cells included a cell population that has previously been described as CFU-F, which has the ability to adhere to tissue culture plastic and proliferate to form a colony composed of the progeny of these founding cells (Ceradini et al. 2004). When the progeny of these progenitor cells are expanded in vitro, they show the same morphology as CFU-Fs derived from human bone marrow.

Recently, novel techniques such as MSC-mediated repair and tissue-engineered bone, ligament, meniscus, and cartilage have been demonstrated (Ohgushi et al. 1989, Bruder et al. 1994, Caplan et al. 1997, Quarto et al. 2001, Buma et al. 2004, Ouyang et al. 2004, Van Eijk et al. 2004, Warnke et al. 2004, Ranieri et al. 2007, Veronique et al. 2007). A stem/progenitor cell fraction from drainage fluid from arthroplasties could be used for postoperative problems such as fractures or revision surgery with bone loss.

The closed suction drainage fluid after hip arthroplasty has direct access to the bone marrow. Thus, multilineage-potential cells (MPCs) can be recruited from the bone marrow. The other possibility is that MPCs may be derived from circulating MPCs. Recently, considerable attention has been paid to the role of accumulating stem/progenitor cells in injured tissue such as thrombus, ischemic zones, and sites of arterial injury (Peled et al. 1999, Weber et al. 2004, Modarai et al 2005). These cells may be involved in tissue repair after surgery. Many authors have suggested that the SDF1/CXCR4 axis plays an essential role in mobilization and homing of stem/progenitor cells (Aiuti et al. 1997, Peled et al. 1999, Ceradini et al. 2004, Kucia et al. 2005). SDF1 is produced in response to tissue damage and plays an important role in the mobilization of CXCR4 positive cells. Several cell types such as cardiomyocytes, muscle-derived fibroblasts, and endothelial cells secrete SDF1. In addition, ischemia and hypoxia can induce SDF1 expression. In the present study, the SDF1 level was found to be increased on the day of surgery and expression of CXCR4 was elevated in the closed suction drainage fluid on the day of surgery and on the following day. This may indicate that locally induced SDF1 levels contribute to the mobilization of circulating CXCR4-positive cells to the injured sites, although direct proof will require further research.

In summary, closed suction drainage fluid on the day of THA contains multipotent stem/progenitor cells that could be expanded by culture in vitro and induced to differentiate via the osteogenic and chondrogenic pathway.
